# Mental and physical outcomes of yoga participation in women with spinal disorders: a qualitative study

**DOI:** 10.3389/fpubh.2025.1685627

**Published:** 2025-10-16

**Authors:** Gamze Akyol, Sermin Ağralı Ermiş, Ömür Fatih Karakullukçu, Özkan Güler, İzzet Karakulak, Cansel Arslanoğlu, Arif Satıcı, Gülşah Sekban, Erol Doğan, Fatma Neşe Şahin, Hamza Küçük

**Affiliations:** ^1^Faculty of Sport Sciences, Aydın Adnan Menderes University, Aydın, Türkiye; ^2^Ministry of National Education, Ankara, Türkiye; ^3^Faculty of Sport Sciences, Ankara University, Ankara, Türkiye; ^4^Faculty of Sport Sciences, Mardin Artuklu University, Mardin, Türkiye; ^5^Faculty of Sport Sciences, Sinop University, Sinop, Türkiye; ^6^Yasar Dogu Faculty of Sport Sciences, Ondokuz Mayis University, Samsun, Türkiye

**Keywords:** women’s health, health promotion, public health, yoga, spinal disorders

## Abstract

Yoga, a holistic practice supporting both physical and mental integrity, has gained increasing attention in recent years as a public health–promoting activity. Particularly among women with spinal disorders (e.g., hernia, lordosis, scoliosis, kyphosis), yoga is recognized not only for improving physical functionality but also for contributing significantly to psychosocial parameters such as stress management, self-awareness, and life satisfaction. In this context, the present study aimed to explore the mental and physical outcomes of regular yoga practice in women diagnosed with spinal disorders, using qualitative methods. This study was conducted using a phenomenological design, as the participants had experienced the situation under investigation. It included semi-structured, in-depth interviews with 15 adult participants diagnosed with spinal disorders and with at least 3 months of yoga experience. In line with the nature of qualitative research, the number of participants was deemed sufficient, and it was accepted that data saturation was reached through the interviews conducted with 15 participants. The findings revealed multidimensional benefits, including mental and physical relaxation, improved stress management, postural enhancement, better quality of life, and increased self-compassion. Participants also highlighted the positive influence of yoga on body–mind balance, awareness of personal limits, and a deeper understanding of their condition. These results suggest that yoga functions both as a physical intervention and a psychological support mechanism for women with spinal disorders, promoting active engagement in their treatment process and enhancing public health outcomes. In conclusion, yoga practice was found to provide significant and sustainable contributions to spinal health and overall psychosocial well-being.

## 1 Introduction

Spinal disorders are a condition seen as chronic musculoskeletal pain. They are characterized by restricted movement (1), loss of working capacity, and health-related complications, all of which pose significant challenges. According to World Health Organisation (WHO) data from 2022, approximately 1.71 billion people worldwide have musculoskeletal disorders (back and neck pain, osteoarthritis, rheumatoid arthritis, etc.). This condition affects people of all ages and from all world regions (2). Furthermore, musculoskeletal disorders are a type of disease that requires rehabilitation on a global scale. Musculoskeletal diseases are not contagious but can lead to the development of cardiovascular diseases (3). Accordingly, physical activity plays a critical role in alleviating associated symptoms, maintaining health, and supporting both physical and psychological well-being (4).

Yoga has increasingly been recognized as an effective therapeutic intervention for managing both physical and psychological challenges associated with chronic conditions, including spinal disorders. Mindfulness-based yoga practices—which integrate physical postures, breath control, and meditation—have been shown to stimulate the parasympathetic nervous system (5), thereby reducing stress, enhancing body awareness, and fostering a positive self-concept (6, 7). Researchers also emphasize that yoga improves flexibility, balance, and muscle strength (8). Hence, yoga may be effective in physical recovery and enhancing psychological resilience (9, 10). Theoretically, yoga can be regarded as a practice that promotes a non-judgmental, mindful self, resulting in the development of a more accepting and positive self-perception (6). The adoption of non-pharmacological therapies such as yoga within a multidisciplinary approach by healthcare professionals further amplifies these benefits and contributes significantly to improved quality of life (11). Thus, yoga serves not only as a physical practice but also as a psychological intervention, reducing stress, strengthening immunity, and promoting overall well-being (12).

There is also growing evidence in the literature regarding the use of yoga interventions to improve physical and mental health (13, 14), with systematic reviews supporting its effectiveness for anxiety (15), depressive symptoms (16), and post-traumatic stress disorder (17, 18). Nonetheless, as with all forms of physical activity, yoga carries risks of injury (8). Reports indicate repetitive strain injuries and damage to the neck, shoulders, spine, legs, and knees due to overextension, particularly when yoga is practiced unsupervised or not adapted to individual conditions (19, 20). Therefore, it is essential for professionals recommending yoga to evaluate both its benefits and potential risks.

Numerous studies in the relevant literature have examined the therapeutic effects of yoga on various conditions, including cancer (21–24), gastrointestinal disorders (25), migraines (26, 27), and depression (28). The most commonly employed research design in these studies is randomized controlled trials (29–31), alongside studies that address the therapeutic benefits of specific yoga styles (17, 46). However, there is a clear lack of qualitative research exploring the psychological benefits and embodied awareness fostered by yoga among individuals diagnosed with spinal disorders contexts where participants often engage in reflective and emotionally immersive experiences.

This study is grounded in self-perception theory, which suggests that individuals construct their self-concept by observing and interpreting their own behaviors and experiences (32), and the present study examines yoga’s effects from the perspective of participants’ lived experiences. The primary aim is to qualitatively evaluate yoga exercises’ positive and negative effects on individuals with spinal conditions and emphasize yoga as an effective method for preventing and treating such disorders. In light of the information presented, the current study seeks to investigate yoga’s physical and psychological benefits, specifically in relation to spinal health. The research is limited to individuals who have been diagnosed with a spinal disorder by a specialist physician but who have not undergone any prior surgical intervention. To avoid influencing participants’ thoughts and emotions, no intervention was applied by the researchers. Therefore, the study’s aim and methodology are assumed to be aligned.

## 2 Method

### 2.1 Research design

This study employed a qualitative research method that allows for an in-depth evaluation of the effects of yoga participation among individuals with spinal disorders using a phenomenological design. Rooted in philosophy and psychology, phenomenology is a research approach that aims to analyze the shared experiences of individuals regarding a particular phenomenon. The information obtained from participants’ lived experiences enables the researcher to reach the essence of the investigated subject (33). In this context, through a phenomenological approach, individual perspectives and perceptions regarding yoga practice were explored, and the various effects of yoga participation on spinal disorders were evaluated.

### 2.2 Study group

The study group consisted of 15 adult individuals diagnosed with a spinal disorder and regularly engaged in yoga practice. According to Creswell and Creswell (33), the number of participants in phenomenological research may range between 3 and 10; thus, it was assumed that the sample size in this study was sufficient for data saturation. The sample size was determined by referencing previous studies with a similar research methodology in the literature: Küçükalpelli et al. (34) and Davenport et al. (47). Participants were selected using the convenience sampling method. Frequently used in qualitative and quantitative studies (35), convenience sampling is one of the most common sampling strategies and is often employed when participants are easily accessible (36). However, this sampling method may limit the generalizability of the findings, as participants who are readily available might not represent the broader population of individuals with spinal disorders. The inclusion criteria for participant selection were as follows: having at least 3 months of yoga experience; having received a medical diagnosis of a spinal disorder (e.g., hernia, lordosis, scoliosis, kyphosis) through physician-conducted examinations and tests; and having no history of spinal surgery. Additionally, since the study focused on both physical and mental outcomes, the presence of clinical conditions such as schizophrenia, bipolar disorder, depression, or severe anxiety was determined as an exclusion criterion. Demographic information of the participant group is presented in Table 1.

**Table 1:** Demographic characteristics of the participants.

n	Age	Type of Disorder	Cause of Disorder	Madication Usage	Duration of Yoga Practice
1	40	Hernia	Heavy lifting, Sedentary lifestyle	Cortisol injection, Painkillers	7 y
2	63	Hernia	Incorrect movement	Painkillers	5 m
3	65	Hernia	Incorrect movement	Painkillers	20 y
4	53	Hernia	Heavy lifting	Muscle relaxant	15 y
5	47	Hernia	Heavy lifting	No medication	6 m
6	36	Lordosis	Genetic factors	No medication	7 m
7	24	Lordosis	Heavy lifting	No medication	6 m
8	23	Scoliosis	Genetic factors	Painkillers	6 y
9	30	Kyphosis	Sedentary lifestyle	No medication	6 m
10	34	Hernia	Sedentary lifestyle	No medication	3 y
11	26	Lordosis	Over weight	No medication	4 y
12	36	Hernia	Heavy lifting	No medication	3 m
13	27	Scoliosis	Sedentary lifestyle, Incorrect movement	No medication	6 m
14	35	Scoliosis	Sedentary lifestyle	No medication	5 m
15	40	Kyphosis	Postural disorder	No medication	1 y

### 2.3 Demographic information

The table above presents demographic data of the participants, including age, spinal disorder diagnosis, reason for surgery, medication use, and duration of yoga participation. Based on this data, the participants’ ages ranged from 23 to 65 years, and their spinal disorders consisted of herniation (n = 7), lordosis (n = 3), kyphosis (n = 2), and scoliosis (n = 3). It was concluded that the primary causes of their conditions were generally heavy lifting and a sedentary lifestyle. However, genetic factors, excess weight, and poor posture were also contributing factors. Pharmacologically, most participants reported not using any regular medication, while those who did use painkillers, cortisol injections, and muscle relaxants.

A wide variation was observed in the duration of yoga participation among the participants. Their involvement in yoga ranged from 3 months up to 20 years, indicating that they had started their yoga practice at different times.

### 2.4 Data collection

Data were collected using a semi-structured interview technique. Semi-structured interviews balance the rigidity of fully structured interviews and the flexibility of unstructured interviews, positioning themselves between these extremes (33). Each interview lasted approximately 45–60 min and focused on questions designed to elaborate on the participants’ yoga experiences.

The data collection process was conducted at a predetermined interview centre for each participant to ensure similar conditions. A total of six questions were administered, aiming to understand individuals’ motivations for practising yoga, the physical and emotional changes they experienced, and the effects of their yoga practice on their spinal disorders. The limitations and findings informed the formulation of the questions of previously published relevant studies in the literature. The questions used during the data collection phase are presented in Table 2.

**Table 2:** Questions used in the data collection phase.

Questions
1. What does yoga mean to you, and what led you to practice it?
2. What effects has yoga practice had on your spinal condition, and what specific changes have you observed (if any)?
3. How has participating in yoga influenced your daily quality of life?
4. What aspects do you pay attention to while practicing yoga, and why?
5. Did your awareness of your spinal disorder increase after starting yoga?
6. How has yoga participation affected your mental health?

### 2.5 Data analysis

In the analysis of the obtained data, the content analysis method was employed, following the steps proposed by Yıldırım and Şimşek (48): coding the data, creating themes, organizing codes and themes, and interpreting the findings. Before the data analysis, a literature review was conducted on spinal disorders and yoga practices to identify perceptions and related concepts among individuals with spinal disorders regarding yoga. Based on this review, the principal codes required for content analysis were defined as spinal disorders and physical effects, mental health, and general perception of yoga.

Subsequently, all collected data were analyzed, and codes that fell outside the scope of the analysis were excluded from the study. As a result of the analysis, a codebook was created, serving as evidence of replicability and consistency, which are considered important for increasing the validity of qualitative data (48, p. 256). The code book’s flows were effectively compared using content analysis results from all independent studies. This ensures that students’ data leads different researchers to the same conclusion, thus ensuring the reliability of the information. The coherence of the previously identified themes was verified using this codebook, and it was assumed that data saturation had been achieved in the research. All data were analyzed using the developed codebook, and the results are presented in the findings section of the study.

To ensure the validity of the research, a strategy commonly used in qualitative studies was adopted: providing and reporting a detailed explanation of all research processes (48, p. 270).

## 3 Results

Results of content analysis from face-to-face interviews with individuals with spinal disorders engaged in yoga practice.

The Table 3 presents the frequency of perceived benefits expressed by individuals with spinal disorders following their participation in yoga, structured around specific thematic codes. The codes represent the most frequently mentioned benefits by participants, and the frequency values (f) reflect the relative importance of these themes within the study group.

**Table 3:** Codebook.

No.	Code(s)	*f*
1	Mental and physical relaxation	21
2	Self-awareness	21
3	Coping with stress	15
4	Psychological and physical health	10
5	Postural improvement and flexibility	10
6	Peace and happiness	9
7	Reduced pain	9
8	Quality of life	6
9	Self-love	5

The codes “mental and physical relaxation” (*f* = 21) and “self-awareness” (*f* = 21) emerge as the most emphasized themes, indicating that participants experience a high level of need or perceived benefit in these areas. Similarly, the code “coping with stress” (*f* = 15) stands out as a significant theme among participants.

The themes “psychological and physical health” (*f* = 10) and “postural improvement and flexibility” (*f* = 10) reflect a balanced expression of participants’ needs related to both mental and physical well-being.

The codes “peace and happiness” (*f* = 9) and “reduced pain” (*f* = 9), though associated with lower frequencies, still represent important physical and emotional needs that contribute to an improved quality of life.

Finally, the emergence of the codes “quality of life” (*f* = 6) and “self-love” (*f* = 5) not only supports the interpretation of other identified codes but also indicates that practising yoga contributes positively to individuals’ overall life satisfaction and fosters a more profound sense of self-love and awareness in coping with spinal conditions.

The results of the content analysis on the theme of spinal health and physical effects in individuals with spinal disorders who participated in yoga practice are presented in Table 4. Among the physical effects of yoga, the most frequently reported benefits by participants were increased mobility (*f* = 6) and reduced pain (*f* = 6). Postural improvement (*f* = 4) and increased flexibility (*f* = 3) were also highlighted as significant findings. Participant statements frequently emphasized the importance of following the instructor’s guidance and paying attention to proper posture during practice. To provide evidence supporting the findings, selected participant responses are presented below:

**Table 4:** Content analysis of the theme of spinal health and physical effects.

Theme	Categories	*f*	Participants
Physical Effects of Yoga Practice on Spinal Health	Increased mobility	6	P4-P1-P15
	Reduced pain	6	P11-P3-P6-P10-P12-P13
	Postural improvement	4	P8-P9-P15
	Increased flexibility	3	P3-P4-P5

P1: “I continue my exercises regularly and with discipline. When I take a break due to life circumstances, I can feel my body becoming stiffer and heavier. When I engage in exercise and yoga practice, I feel more relaxed and liberated. Above all, yoga enhances one’s quality of life—it is not solely a physical or mental experience but rather a discipline that harmoniously blends both. It helps with mental relaxation, emotional regulation, and acting with greater awareness, allowing you to live more consciously.”

P6: “Since I also practice yoga at home or elsewhere in daily life, it relaxes me tremendously, and I feel my spine stretching. When my pain increases during the day, I do yoga poses and the pain decreases. Because of the issue in my foot, I try to be careful not to make any wrong movements. That’s the main aspect I pay attention to.”

P8: “Yoga has had a significant impact on my daily life. I’ve observed major improvements in my posture, and people around me have also noticed the change. While practicing yoga, I pay particular attention to the poses. Since the spine is a focal point, I’m especially mindful of proper stretching.”

P11: “For me, yoga represents both mental and physical relaxation. It helps clear my mind and lowers my stress levels during the day. My yoga journey began during my fourth year of university with a yoga course. Before that, I used to feel lower back pain during long walks, but after starting yoga, especially my lower back pain decreased, my walking duration increased, and the discomfort subsided.”

P13: “Due to my condition, I had intense back and lumbar pain, which still persists to some extent. However, after yoga classes, I feel my muscles softening and a sense of relief. I pay special attention to movements that target the back and spinal areas, and I also closely follow the instructor’s guidance.”

The results of the content analysis regarding the effects of yoga practice on mental health in individuals with spinal disorders are presented in Table 5. Participants strongly expressed the positive impact of yoga on mental well-being. The most frequently emphasized benefit was mental relaxation/calmness (*f* = 5), followed by coping with stress (*f* = 6). In addition, some participants stated that they developed more positive thoughts (*f* = 3).

**Table 5:** Content analysis for the theme of mental health and yoga.

Theme	Categories	*f*	Participants
Mental Health and Yoga	Mental relaxation/calmness	5	P2, P6, P7, P9, P11
	Coping with stress	5	P1, P7, P10, P11, P14, P15
	Positive thinking	3	P9, P14, P15

To support the categories formed based on the findings, sample participant statements are presented below:

P7: “For me, yoga is complete relaxation. While practicing yoga, I feel refreshed and mentally very relaxed. I was introduced to yoga by a friend who has the same condition as mine. Since I work in a highly intense and stressful work environment, I release all my stress when I come to yoga sessions. I pay close attention to my instructor’s guidance during practice and focus on doing the movements correctly. Initially, I attended for physical health, but I later realized it had a great impact on my mental well-being, which became my main focus.”

P9: “I started yoga upon my doctor’s recommendation. Yoga helps me clear my mind and find inner peace. It also helps me love myself more and escape from pessimism. It improved my posture and corrected my spinal misalignment. I can say that it has greatly relaxed me mentally. During practice, I pay close attention to the instructor’s cues and make sure I perform the postures correctly.”

P11: “Yoga provides a very positive effect by regulating my stress levels and relaxing my muscles. While doing yoga, I’m careful not to put too much strain on my lower back and try to stay within the limits of my body. It has greatly benefited my mental health. Before yoga, I could never silence the thoughts in my mind—but after starting yoga, I realized that I could quiet those internal voices.”

P14: “When I begin a yoga session, I feel that both my body and mind completely relax, and this makes me very happy. In daily life, I can think from different perspectives and more calmly and clearly. I start my day more energetic and cheerful. My communication and attitudes toward others have improved significantly. I used to have very little patience, but now I feel more emotionally developed thanks to yoga.”

P15: “Participating in yoga has significantly improved my quality of life. I feel more energetic and balanced both physically and mentally. I wake up more easily in the mornings and stay more focused and patient throughout the day. Yoga has taught me to listen to my body and accept my limits, which has made me more compassionate toward myself. Additionally, dealing with stress has become easier; thanks to the breathing techniques I’ve learned, I can stay calm even in difficult situations. In short, yoga has made my life more balanced, peaceful, and fulfilling.”

The results of the content analysis related to the theme of perception of yoga among individuals with spinal disorders who participated in yoga practice are presented in Table 6. Participants’ perception of yoga is primarily shaped by mind–body balance (*f* = 5). They perceive yoga as a physical activity and a holistic discipline promoting physical and mental harmony. Other key categories include discovering personal limits (*f* = 4), instructor-centered practice (f = 4), and correct posture implementation (*f* = 3), which highlight the participants’ conscious and mindful approach to yoga.

**Table 6:** Content analysis of the theme: perception of yoga.

Theme	Categories	*f*	Participants
Perception of Yoga	Mind–body balance	5	P1, P6, P10, P14
	Discovering personal limits	4	P1, P9, P15
	Instructor-centered practice	4	P2, P3, P10, P15
	Correct posture implementation	3	P8, P15

To provide supporting evidence for the categories derived from the findings, participant quotations are presented below:

P1: “Yoga, for me, is a means of relaxation as well as an exercise for both body and mind, contributing to a positive resting and calming process. It was something I had always wanted to learn, and I began my yoga journey as soon as I found the opportunity. What I pay attention to most is my own body and its limits. Yoga is not about striving to achieve a perfect pose or competing with others—it’s about discovering your own physical boundaries. That journey of self-discovery never ends, and as a result, the process of personal development continues endlessly. The more you practice, the more you can evolve.”

P14: “Yoga represents a union of body and mind for me. I started yoga upon a friend’s recommendation. I pay very close attention to performing the poses and exercises correctly and avoid any movement that might be harmful due to my condition.”

P15: “Yoga practice had incredibly positive effects on my spinal issues, though it took some time for me to realize this. I used to experience unbearable back and lower back pain even during daily activities such as standing or sitting for extended periods. Although I sought physiotherapy, I only found lasting relief through yoga. The practice not only strengthened my spine but also made me aware of the muscle groups that support it. For instance, learning to align properly in each pose helped reduce the load on my spine and gave me a better understanding of how my body functions as a whole. Poses like Cat-Cow, Downward Dog, and Sphinx helped increase spinal mobility while reducing pain, making me feel more flexible and comfortable. I pay close attention to listening to my body’s limits, maintaining correct alignment, and synchronizing movements with breath. This reduces the risk of injury and makes the practice both more effective and enjoyable.”

The content analysis results regarding the impact of yoga participation on awareness of spinal disorders among individuals with such conditions are presented in Table 7. The findings indicate that yoga enhances participants’ awareness of their spinal conditions. The table shows that increased body awareness (*f* = 6) and knowledge and control of the illness (*f* = 5) are the most frequently expressed categories. Only two participants stated that they did not gain any awareness, supporting the notion that yoga functions as an awareness-enhancing tool. Below are selected participant statements provided as evidence for the categories formed based on the findings.

**Table 7:** Content analysis of the effect of yoga participation on awareness regarding spinal disorders.

Theme	Category	Codes	Frequency
Gaining Awareness	Increase in Body Awareness	“I learned how to use my body,” “I understood where the problem is”	6
	Knowledge and Control of Illness	“I became aware of the illness,” “I noticed its benefits”	5
	Contribution of Yoga to Treatment	“My muscles got stronger,” “It supported me after surgery”	4
Fixed Awareness	Fixed Awareness or Unclear Effect	“There was no increase in awareness,” “I only feel relaxed”	2

P1: “It definitely increased my level of awareness. I gained a great deal of knowledge both about the illness and about how I should use my body.”

P8: “My awareness certainly increased. I wasn’t this informed about my condition before, but through yoga I became more conscious, and my awareness level improved.”

P9: “Yes, my awareness of the illness increased significantly. At first, I did not think it would benefit my condition, but after seeing its effects, I started to see it as important.”

P15: “Yes, as I continued practicing yoga, my awareness of my spinal disorder significantly improved. I began to better understand the needs and limits of my body. Realizing which movements provided relief and which caused discomfort helped me manage the condition and reduce pain.”

## 4 Discussion

This study examined the physical and mental outcomes of yoga among individuals with spinal disorders using a qualitative approach. The aim was to evaluate the therapeutic potential of yoga for spinal health and to reveal awareness gains related to the mind–body connection based on participants’ experiences. Through a phenomenological design, in-depth semi-structured interviews were held with 15 participants, and the data were systematically analyzed using content analysis. The findings indicate that yoga provides multidimensional benefits, particularly regarding psychological well-being, physical relaxation, self-awareness, stress management, and postural improvement. Participants reported physical improvements and enhanced mental balance, awareness, and overall quality of life. These outcomes can be interpreted through the lens of self-perception theory, which posits that individuals develop attitudes and self-concepts by observing their own behaviors (32). By engaging in yoga, participants could observe their physical and psychological improvements, thereby reinforcing positive self-perceptions and enhancing motivation for continued practice. These findings suggest that yoga is a complementary approach to managing spinal disorders and promotes individuals’ active involvement in the treatment process by increasing internal awareness.

The qualitative findings of the mixed-method study by Arya et al. (29) also support that yoga enhances both physical and mental well-being and a perceived sense of empowerment and coping capacity among individuals with spinal cord injuries. Similarly, studies by Chalageri et al. (9) and Curtis et al. (30) found that yoga interventions in patients with spinal cord injuries and post-surgical conditions reduced depressive symptoms and perceived pain while increasing self-compassion and mindfulness. Riley and Park (7) and McCall (10) also reported that yoga provides psychological benefits for anxiety, depression, stress, and emotional regulation. From a self-perception theory perspective, these improvements likely reinforce participants’ self-evaluations of their abilities and well-being, contributing to a more positive self-concept and sustained engagement in health-promoting behaviors. Taken together, our findings and the literature suggest that yoga may reduce perceived pain and mental disorder in individuals with spinal disorders while increasing self-compassion, self-awareness, and emotional well-being.

The participants’ age range extended from 23 to 65, indicating that yoga is accessible and beneficial for individuals across different age groups—not just the young. Notably, older participants (e.g., aged 60 and above) reported improved pain control and mobility, demonstrating that yoga can help manage age-related physical limitations. In addition, yoga is an exercise form with dozens of different styles and postures (37), and it is known that most of the research participants practiced Hatha and Vinyasa Yoga. This indicates that yoga and its styles are accessible to individuals of different age ranges and physical characteristics. Cramer et al. (38) support this interpretation with their explanation. According to the researchers, different yoga styles do not differ in their likelihood of yielding positive outcomes in randomized controlled trials; therefore, personal preferences and suitability should guide the selection of an individual’s yoga style. The most common spinal condition reported among participants was hernia (n = 7), mainly attributed to heavy lifting and sedentary lifestyles. However, genetic factors, excess weight, and poor posture were also contributing factors. This group reported improvements in pain management, postural alignment, and mobility following yoga practice, suggesting that yoga may be protective and supportive for such mechanical disorders. Likewise, participants with structural spinal conditions like lordosis, kyphosis, and scoliosis reported positive outcomes from regular yoga practice, including improved body awareness and symptom relief. Crow et al. (46) also stated that yoga may effectively manage neck and back pain. On the other hand, the difference between the study participants in terms of age and diagnosed disease limits the generalizability of the findings, and this should be noted for the current literature. Marotta et al. (39), focusing on women with chronic low back pain, found that yoga postures may positively influence pain intensity and the flexion-relaxation phenomenon. However, 1 month after the intervention, retention test results showed that participants reverted to values close to those of the control group. Ignatova (4) concluded that posture disorders improved in children practising yoga twice a week for a year. These findings imply that the sustainability of yoga practices is essential for long-term benefits in spinal disorders, also recommend the development of yoga interventions tailored to spinal conditions and call for long-term effect studies.

Both short-term (3 months–1 year) and long-term (5 years or more) practitioners experienced similar positive effects. This suggests that yoga can lead to meaningful physical and psychological changes even in a short period. However, long-term practitioners used more profound and conscious expressions about body awareness, stress coping, and mental balance, indicating that continuity in yoga practice deepens its benefits. Self-perception theory explains this pattern: repeated engagement in yoga allows individuals to repeatedly observe their own improvements, which strengthens positive self-concepts and encourages consistent practice. These findings are consistent with those of Tihanyi et al. (40), who found that mindfulness and satisfaction with body image mediated the relationship between advanced yoga practice and psychological well-being. McCall (41) also supports these outcomes, emphasizing yoga’s utility in alleviating psychological distress. Gard et al. (42) proposed a model explaining yoga’s psychological benefits through cognitive, emotional, and behavioral mechanisms alongside physiological ones. The model suggests that yoga fosters psychological improvements by enhancing attention, self-awareness, decentering, reappraisal, and non-judgmental observation. Studies by Bennet (17) and Hendriks et al. (49) further reinforce yoga’s positive effects on psychological well-being.

Another noteworthy finding was that most participants either did not use medication or used it only occasionally (e.g., painkillers, cortisol injections). This suggests that yoga practices reduce the need for pharmacological interventions. From a self-perception perspective, participants’ recognition of their ability to manage symptoms through yoga may reinforce their sense of autonomy and confidence in self-management, contributing to decreased reliance on medication. Regular yoga practice appears to be a supportive alternative, particularly for pain management and muscle relaxation. However, researchers did not directly control the yoga practices in this study, and the limited sample size and the sample group is Türkiye-based constrains generalizations, particularly regarding pharmacological outcomes. McCall et al. (43) emphasized that yoga can be a safe and supportive mind–body practice, functioning as an adjunctive therapy for spinal or spinal cord-related conditions. Still, one key implication of the current study is the need for instructor-guided, posture-appropriate practices, especially for individuals with chronic conditions, to mitigate potential risks. Awan et al. (8) also highlighted that expert practitioners should guide yoga poses to maximize benefit. Sujatha et al. (44) noted that yoga may enhance physical strength, detoxification, circulation, and mental balance when used alongside pharmacological treatments. Similarly, Crow (46) indicated that properly designed yoga practices could reduce pain and improve functionality as a non-pharmacological treatment method. These findings align with the current study’s conclusions. Thus, yoga practices should be customized to the individual and their medical history and performed under professional supervision, particularly for patients with specific health conditions.

## 5 Conclusion

The primary aim of this study was to qualitatively assess the physical and psychological outcomes of regular yoga practice among individuals with spinal disorders and to explore its therapeutic potential. Since spinal disorders negatively affect physical functioning and quality of life, the scientific evaluation of such complementary practices is critical. Findings from this phenomenological study suggest that yoga provides physical benefits such as pain reduction, improved posture, and enhanced mobility but also contributes significantly to psychosocial aspects such as self-awareness, stress management, mental relaxation, and life quality. Participants reported enhanced mind–body awareness, increased illness-related consciousness, and reduced reliance on medication. These results may enable yoga to be evaluated as a complementary intervention in the treatment of spinal disorders.

However, certain limitations of this study must be acknowledged. The small sample size, the lack of researcher-led yoga sessions, and the absence of a controlled intervention program limit the generalizability of the findings. Furthermore, excluding individuals with surgical histories means these results may not directly apply to such populations. Therefore, future research should involve larger samples, include controlled intervention programs, and compare spinal disorders. Longitudinal studies evaluating the sustainability of yoga’s effects and its integration with pharmacological and psychological therapies would also contribute to the literature. For practitioners, it is crucial to design yoga practices based on individual differences, health histories, and physiological limitations. For professionals, yoga may be recommended as a holistic approach that complements pharmacological treatments and enhances quality of life. Accordingly, yoga can be viewed as a tool for physical recovery and a powerful therapeutic method that meaningfully supports self-awareness, psychological well-being, and life satisfaction.

## Data Availability

The raw data supporting the conclusions of this article will be made available by the authors, without undue reservation.
